# Supplementary material for: Chemical unclonable functions based on operable random DNA pools
Source: Nat Commun. 2024 Apr 5;15:2955. doi: 10.1038/s41467-024-47187-7 (PMC10997750; doi:10.1038/s41467-024-47187-7)
Supplement: Supplementary file 1 — Supplementary Information [file 41467_2024_47187_MOESM1_ESM.pdf]

SUPPLEMENTARY INFORMATION TO THE MANUSCRIPT

CHEMICAL UNCLONABLE FUNCTIONS BASED ON OPERABLE RANDOM DNA POOLS

Anne M. Luescher[1], Andreas L. Gimpel[1], Wendelin J. Stark[1], Reinhard Heckel[2] und Robert N. Grass[1]*

[1] Department of Chemistry and Applied Biosciences, ETH Zürich, Vladimir-Prelog-Weg 1-5, 8093, Zürich, Switzerland

[2] Department of Computer Engineering, Technical University of Munich, Arcistrasse 21, 80333, Munich, Germany

*Corresponding author, robert.grass@chem.ethz.ch

Supplementary Information

Supplementary Figures 1-17

Supplementary Notes 1-13

Supplementary Tables 1-5

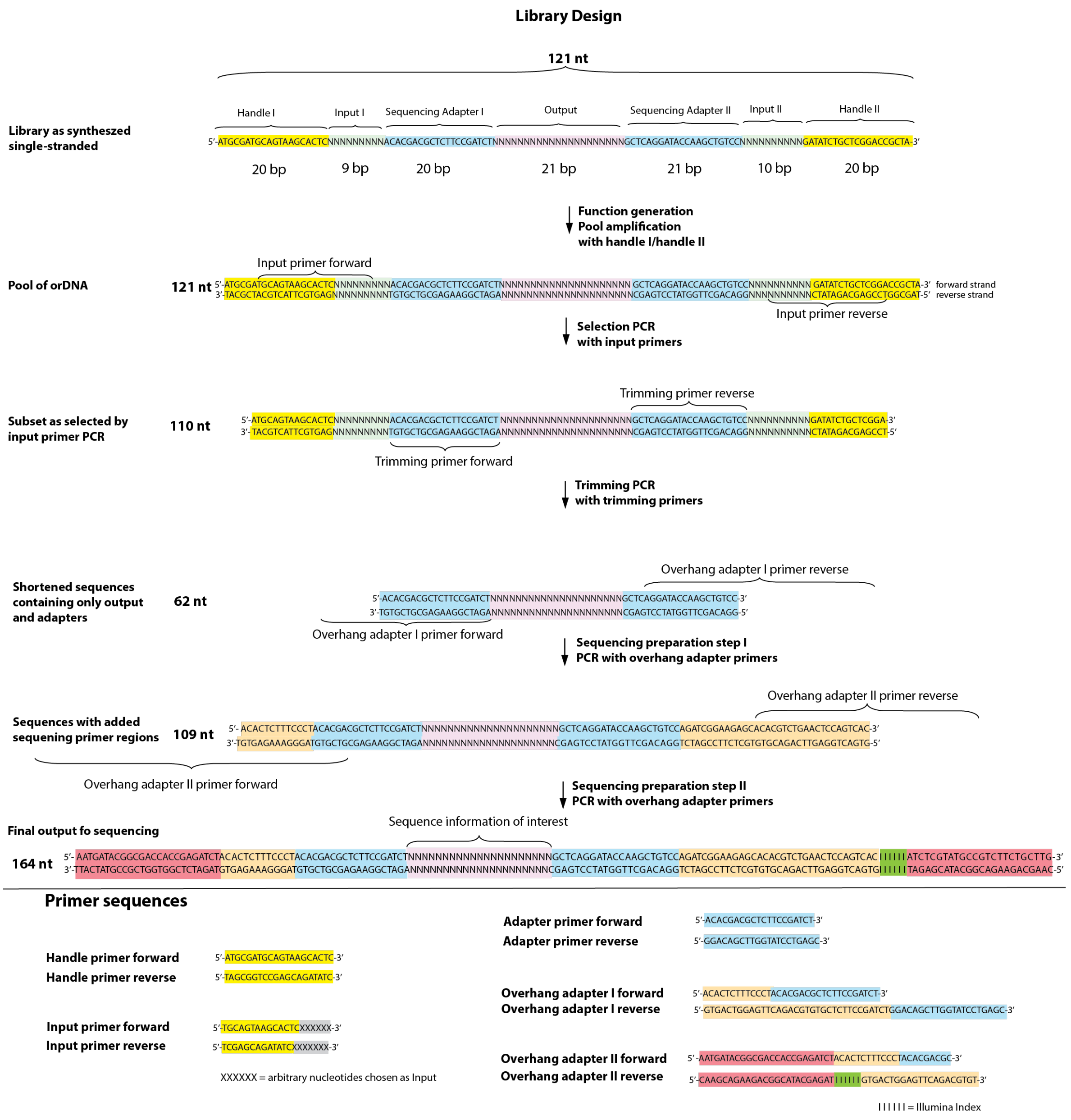

Supplementary Fig. 1: Library design

Shown are the sequence structures at various stages of the process of creating and operating the chemical random function. First, a pool comprising several milligrams of single-stranded oligonucleotides is obtained from solid-state synthesis, comprising both sequence-determined and random segments. A subset of this library (e.g. 10^8 – 10^{11} sequences) is then amplified via PCR using the outer handles as primers. The orDNA pool can then be operated using a set of input primers. The two inputs comprise a few bases (number may vary between 6 and 9), which selectively bind to the sequences in the pool with complementary matching segments in their randomly synthesized regions. Aside from the bases binding to the input regions, the primer further contains a part of the handle sequences to guide the input towards the correct binding position and to add enough length to the

primers for successful amplification. The selected sequences are only a very small subset of the orDNA pool and are identified via next generation sequencing. To prepare sequencing, three further PCR reactions are conducted. First, the sequences are trimmed using the two adapter primers. The vast majority of the resulting pool then only contains the two adapters with the output section in between. In two subsequent steps the Illumina sequencing overhang adapters are introduced, which then allow readout of the output. Representative gel images of the different stages can be found in Supplementary Fig. 4.

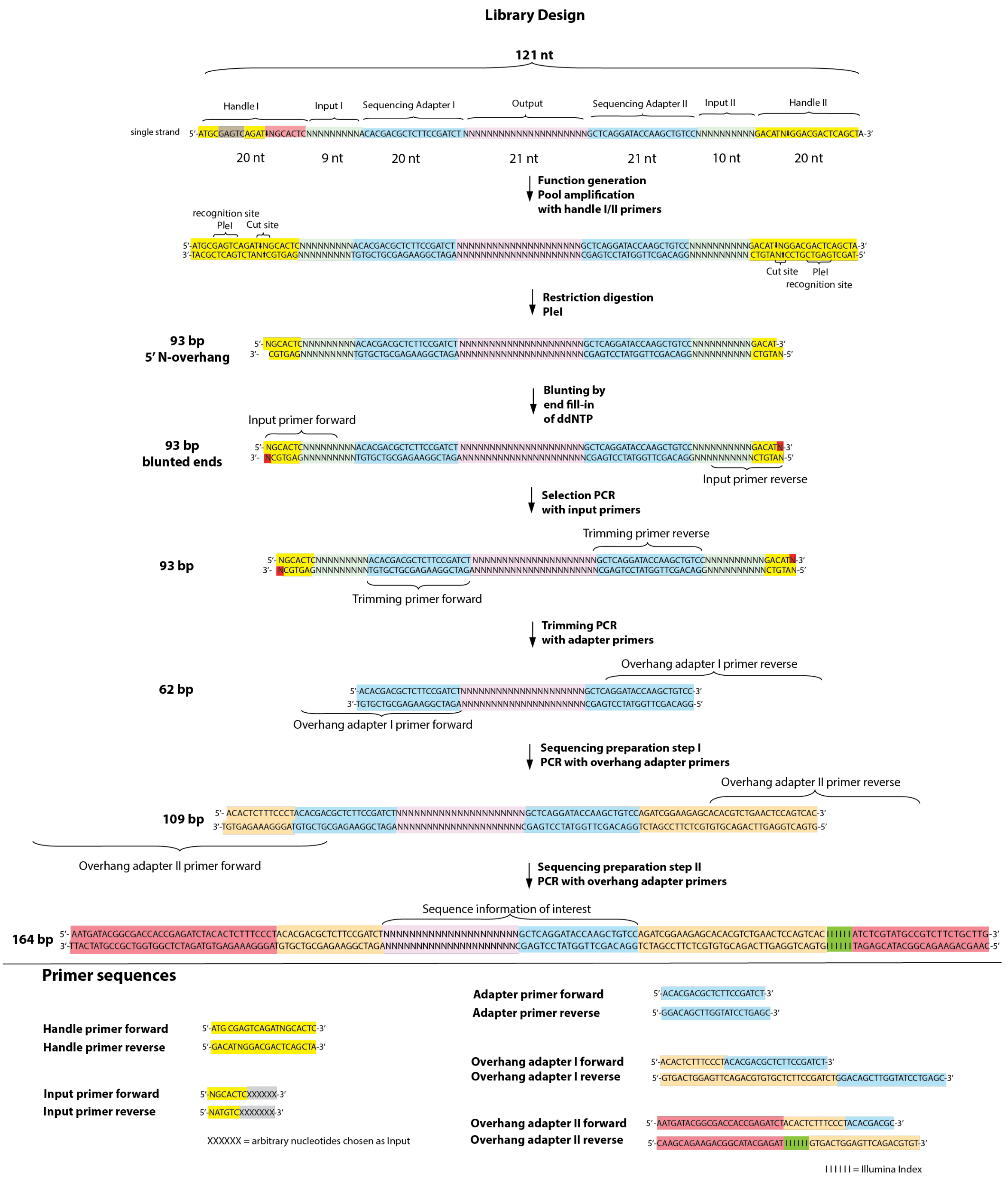

Supplementary Fig. 2: Library design with cleavable handles.

Shown are the sequence structures at various stages of the process of creating and operating the chemical random function. The working principle is identical as described for the library described in Supplementary Fig. 1, but there are additional steps to make the orDNA not only operable, but unclonable. The double-stranded (ds) PCR pool is treated with a type IIS restriction enzyme, PleI, that removes a large part of the handle and leaves a 5’ overhang of a degenerate nucleotide. The sticky

end is blunted using Sequenase, which incorporates the complementary nucleotide with a dideoxy modification.

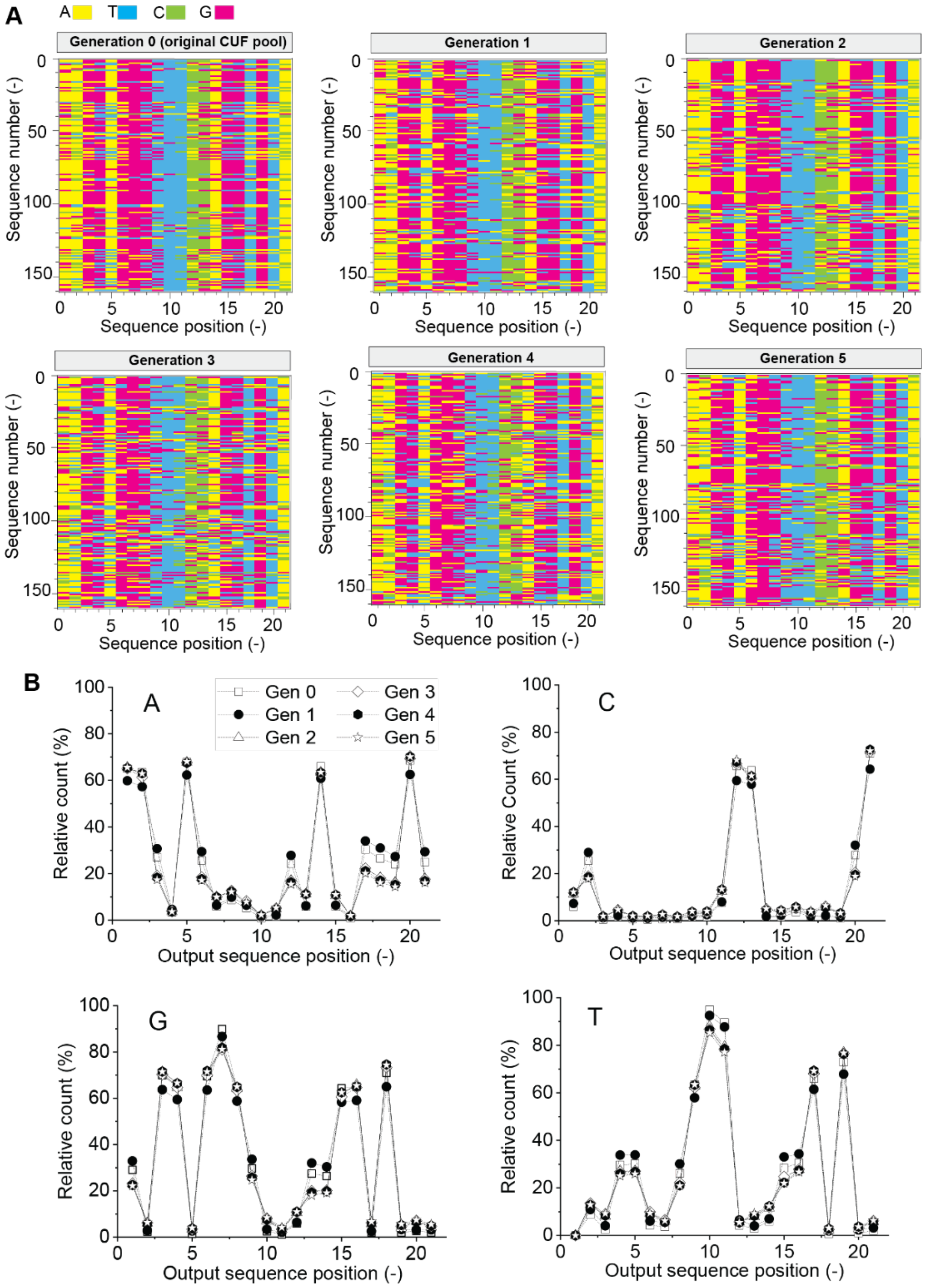

Supplementary Fig. 3: Raw data analysis of proliferation CRPs

Position-dependent raw data analysis of experiments 1 and 6-10, comprising the Illumina sequence reads resulting from the same input applied to five generations of the same CUF, i.e. the same orDNA pool, showing a high qualitative similarity between all generations (also refer to Fig. 3 (b), (c)). Each generation was created by copying the previous generation using PCR. A) Color-coded visualization of the occurrence of the four nucleobases A, C, G and T across the 21 positions of the output sequences. Shown are the first 160 reads out of the respective FASTQ file after filtering for the

presence of the constant adapter regions. The selection is thus arbitrary without any applied order. B) Relative frequency of the four nucleobases A, C, G and T across the 21 positions of the output across the entire sequence set resulting from Illumina sequencing. Source data are provided as a Source Data file.

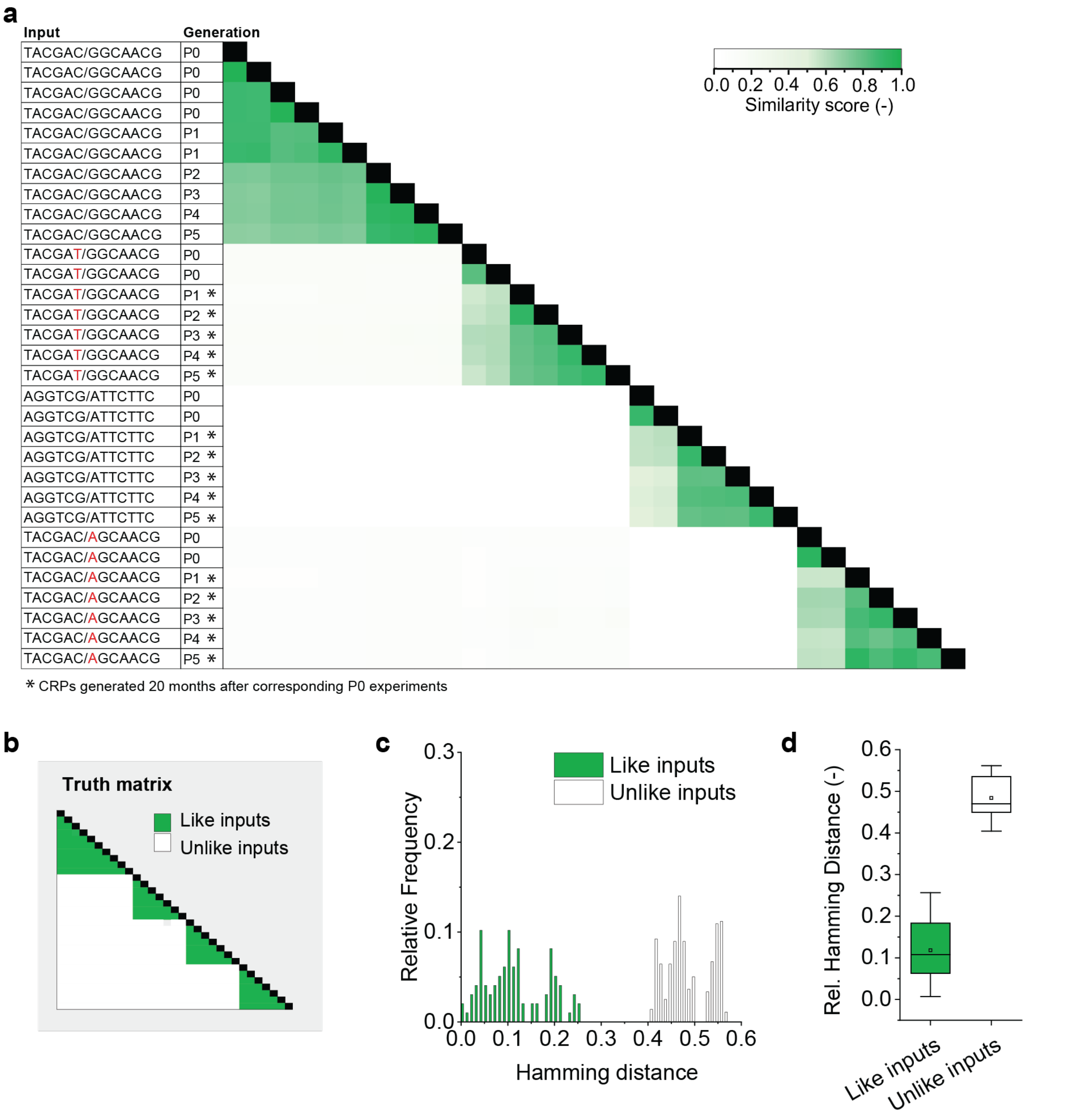

Supplementary Fig. 4

Extended data for generations P0-P5 (experiments 40-54, refer to Suplementary Tables 4-5), using four different inputs. Inputs 2-4 with generations P1-P5 were measured 20 months after the CRPs generated with P0. **a** shows the Jaccard similarity of CRPs comparing P0-P5 outputs. Jaccard similarity across generations as well as the 20-month time gap among like inputs is still well above Jaccard similarity of any CRP generated with unlike inputs, including the ones differing by a Levenshtein distance of 1. **b** Corresponding truth matrix to (a). **c** Histograms of Hamming distances after MinHashing showing the distributions of like and unlike inputs for experiments as shown in (a). n = 357 comparisons for unlike distribution, n = 98 for like distribution. **d** Boxplot showing average

relative Hamming distances, as per the distributions in (c). Indicated are the median (middle line), mean (circled dot), 25th and 75th percentile (box) and 1.5 interquartile range (whiskers), with outliers marked as black dots. n = 357 comparisons for unlike distribution, n = 98 for like distribution. Source data are provided as a Source Data file.

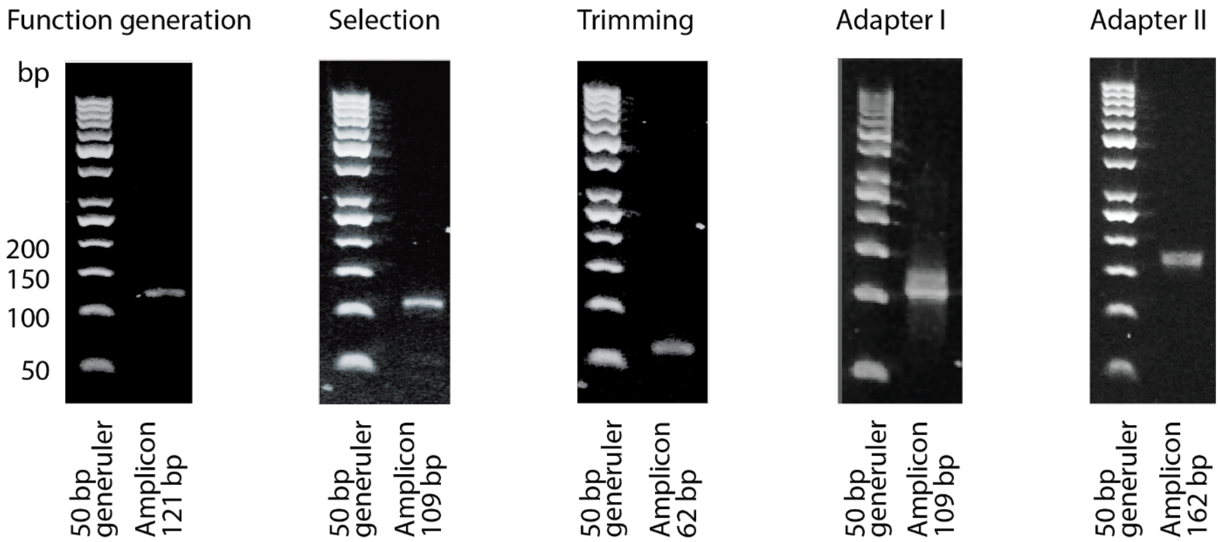

Supplementary Fig. 5

Agarose gel electrophoresis photographs of the different steps of function generation and operation, showing all stages of experiment 1. Images were converted from color to greyscale and edited in brightness and contrast using Adobe Photoshop.

Supplementary Note 1: Random DNA synthesis

Solid-support synthesis of DNA is commercially widely available. In standard chemical synthesis of DNA, the strands are grown nucleotide by nucleotide in an iterative reaction, where each cycle consists of several steps and adds a single base to each growing chain (Fig. 5). For most use cases, this is done in a controlled manner, meaning only one type of building block (dNTP) at a time is added to achieve billions of identical strands of constant length and known sequence. However, it is possible to instead add mixtures of the four nucleobases A, C, G and T. In this case each growing chain will incorporate only one of the four possible building blocks. It has been shown that this process is random[1], meaning the entropy of a mixture can be exploited in a chemical reaction resulting in DNA molecules of random sequence. Each sequence can thus be understood as a random (binary) number, where every base contributes two bits. As the synthesis is stepwise, it can be individually decided for each position along a synthesized sequence whether that specific position will contain a determined or a randomly incorporated building block.

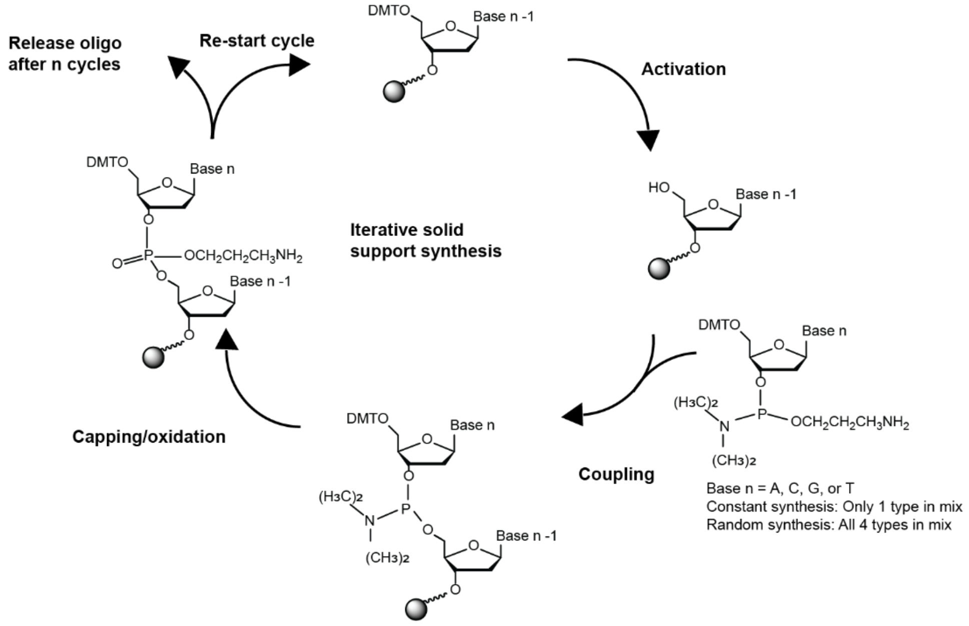

Supplementary Fig. 6: Schematic representation of chemical DNA synthesis.

The cyclic process adding a single nucleotide to each growing chain consists of an activation step, followed by coupling, capping/oxidation. The cycle is followed until the desired chain length is achieved, after which the synthesized oligonucleotide is released from the solid support. In the case of sequence-determined synthesis, only a single type of nucleotide is added during the coupling step,

while for random synthesis, all four building blocks are mixed previous to addition, with each growing chain incorporating only one of the four.

Supplementary Note 2: Unpredictability and unknowability of sequence composition

As in chemical unclonable functions each DNA strand contains segments that are randomly synthesized, given the stochasticity of the process, it is a priori impossible to know the composition of any given strand. For a chemical unclonable function based on operable random DNA this unpredictability is key, as it translates to a random combination of input and output sequences. Therefore, a given output sequence does not confer any information about the base composition of the input and vice versa (see also Supplementary Note 3).

Supplementary Note 3: Expected distribution of sequences within random pools and pool entropy

There are 4^n possible sequences for a randomly synthesized batch of DNA, where *n* corresponds to the number of synthetic cycles using a dNTP mixture. The cycles using a single dNTP type, i.e. the cycles for synthesis of the sequence-determined parts, are not included in the calculation, as they do not contribute to the possibility space (aside from a negligible contribution by errors). As in this work, libraries with a total of 40 random positions in the input and output segments were implemented, there are 4^{40} = ca 10^{24} possible sequences, corresponding to approx. 1.66 mol of DNA. With an average of 330 g/mol/base·121 bases (random and constant combined) this corresponds to a molecular weight of ca. 40’000 g/mol for the single-stranded synthesis product of our library. This means to synthesize every possible combination on average at least once, ca. 40’000 g/mol·1.66 mol = 66 kg of DNA would have to be produced. Around 4 mg of DNA were synthesized in total, equivalent to ca. $6 \cdot 10^{16}$ sequences. Importantly, the synthesis process only comprises individually growing chains without copies (the sequences are only copied post-synthetically in a PCR reaction). This means any duplicates only exist by chance and not by design. The probability for a specified pair of two individual sequences being the same equals to $0.25^{40} = 8.27 \cdot 10^{-25}$.

From the synthesized pool, 10^8 sequences, $1.6 \cdot 10^9$ and $2.6 \cdot 10^{10}$ sequences were arbitrarily extracted to generate the three CUF sizes (with prefixes S, M and L). The extraction was performed by dissolving the dried pool in ultrapure water to a known concentration. Using dilution series, it is then possible to draw a volume that approximately corresponds to the number of desired sequences, which are then subjected to PCR for function generation. With this ratio of function-determining vs. possible combinations, the vast majority of the sequences is expected to be unique, with the overall average copy number being very close to one.

As there are four possible building blocks per randomly synthesized sequence position, two bits of entropy are encoded in a single base (translating to assigning binary numbers 00, 01, 10, 11 to the four bases). As there are 40 random bases per sequence, this corresponds to 80 bits of random information. As established above, ca 4 mg ($6 \cdot 10^{16}$ sequences or 100 nmol) of an orDNA library were synthesized by a commercial supplier. This results in a total entropy of 80 bits/sequence x $6 \cdot 10^{16}$ sequences = $5 \cdot 10^{18}$ bits = 0.5 Exabyte (rounded to the first decimal position) in the pool.

Supplementary Note 4: Design considerations for input primers

Not all conceivable PCR primers are suitable input primers. Most importantly, the ratio between the function size (= number of unique DNA sequences in a selection PCR, e.g. 10^8) and the chosen input length in base pairs is relevant: If the input is too long, a given primer may not find any complementary sequence in the pool to bind to, leading to an unspecific signal, or no amplification at all. If it is too short, too many sequences may be amplified for successful signal extraction. This relationship is shown in Fig. 6, which plots the expected number of output sequences against the input length for three different pool sizes. For the pilot experiments, the input length was selected such that the expected number of sequences that perfectly match to a given input primer sequence in the pool is approx. 1.5. The actual number of matches varies between experiments and is expected to be Poisson-distributed. For a pool of 10^8 sequences, the input length corresponding to this value is 13 random bases in total ($4^{13} = 6.7 \cdot 10^7$). The input was distributed over the forward and reverse primers with 6 and 7 input bases, respectively. For the primers to reach high enough melting temperatures, 14 additional nucleotides were added, which were chosen to overlap with the respective outer handle sequences (see Supplementary Fig. 1). This partial overlap with the constant regions of the orDNA is additionally desirable, as this encourages binding at the intended positions, i.e. the random input segments and not the random output segment. This results in amplicons of constant length and ensures the amplified sequences contain the output segment, which is the chemical readout of the function necessary to calculate a valid output.

However, inputs with a higher number of selective nucleotides have been shown to still generate reproducible outputs in the test setting, even though no perfect counterpart is expected to be present in the pool. This can be explained by the fact that there are always primer-template hybrids that are thermodynamically more favored than others, and the corresponding sequences are thus favorably amplified, even if the match is imperfect. As the readout of the function is generated by the entirety of the amplified sequences and their frequency of occurrence, this distribution still works as a reproducible signature. However, with an increased input length, a lower proportion of the nucleotides is expected to contribute to the specificity of the readout and the noise increases, potentially leading to the occurrence of collisions with shorter inputs. Therefore, depending on the application scenario, it is advantageous to restrict the input length to conform to the respective pool size (see also Supplementary Note 8.

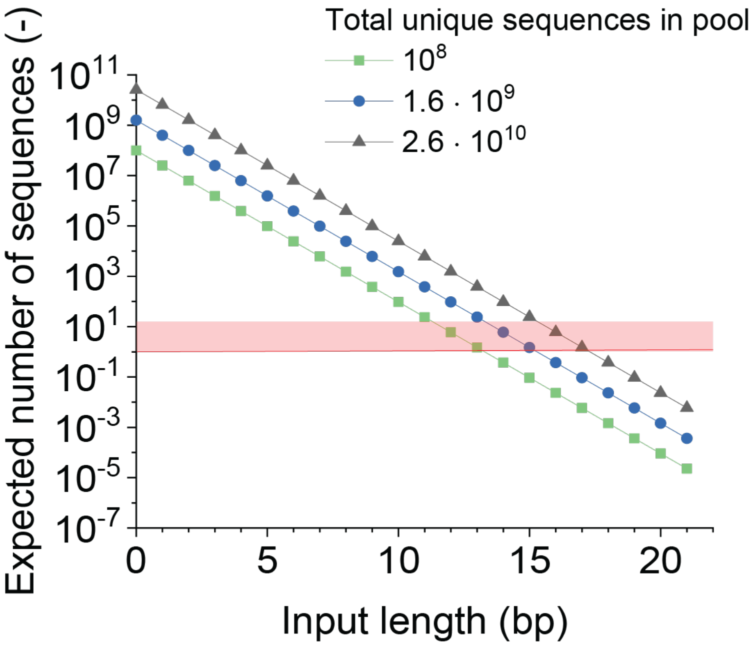

Supplementary Fig. 7

Relationship of the expected number of matching output sequences in a pool related to the input length in basepairs (bp) at different orDNA pool diversities. The red area approximately marks the range this work operated in. Source data are provided as a Source Data file.

Supplementary Note 5: Filtering of the sequencing reads

Before applying k-mer extraction and computation of similarity, the reads were filtered to remove any artefacts and potential contaminations. All correctly amplified orDNA sequences are expected to have the same overall arrangement of their constant and random regions. Therefore, using the BBmap (v38.99) command below, the reads were filtered for the presence of the constant primer region, allowing a maximum Hamming distance of 3. Of the reads passing the first filter, those were excluded that did not contain a 21-bp long insert between the expected constant segments. Only these inserts of the passing reads, comprising the randomly synthesized ‘output’ portions, were included for further analysis.

```
bbduk.sh in=<input.fq.gz> outm=stdout.fq ref=primer.fasta k=21 hdist=3 | bbduk.sh in=stdin.fq
out=<output.fq.gz> ref=primer.fasta ktrim=r interleaved=f k=21 hdist=3 maxlength=21 mininsert=21
minlength=21
```

Supplementary Note 6: Choice of k-mer extraction and Jaccard similarity as data processing methods

In contrast to mathematical operations, data collected from real-world experiments are noisy. For the assessment of chemical unclonable functions, this is an issue, as two individual operations of the function will almost never lead to the exact same result, even if the equivalent inputs were used. Physical unclonable functions and biometric data processing, e.g. human fingerprint analysis, face the same issue. As a consequence, such datasets are analyzed in terms of their similarity instead of perfect identity, with a defined similarity threshold above which the noisy datasets are considered identical.

A k-mer-based method is suitable, as k-mers are commonly utilized in bioinformatics and, more specifically, applied for genome comparisons[2]. As the sequences of interest (the ‘output’ segments of the sequences amplified during PCR selection with the input primers) are only 21 nt long, a circular k-mer extraction was applied. This approach can be described by virtually circularizing the sequence of interest and forming k-mers around the circle, as explained in Supplementary Fig. . For a sequence of length *n* this leads to *n* k-mers independently of *k*, while linear extraction only yields *n*-*k*+1 k-mers. Circular analysis therefore puts equal weight on all sequence positions, while a linear analysis would over-emphasize the center nucleotides relative to the edges.

Further, to compare the k-mer sets extracted from the sequencing data, Jaccard similarity was used. This index is widely used in machine learning, computational genomics and other fields [3]. It is calculated by the ratio between the size of the intersection of two sets *A* and *B* divided by their union:

$$\mathrm{J}(A,B) = \frac{|A \cap B|}{|A \cup B|}$$

Furthermore, the Jaccard coefficient can be weighted:

$$\mathrm{J}(A,B) = \frac{\sum_k \min(A_k, B_k)}{\sum_k \max(A_k, B_k)}$$

While the unweighted coefficient only considers the presence or absence of a given k-mer, the weighted coefficient considers the frequency of occurrence and thus the contribution of each k-mer to the overall ‘fingerprint’. Applying a weighted coefficient is therefore more comprehensive.

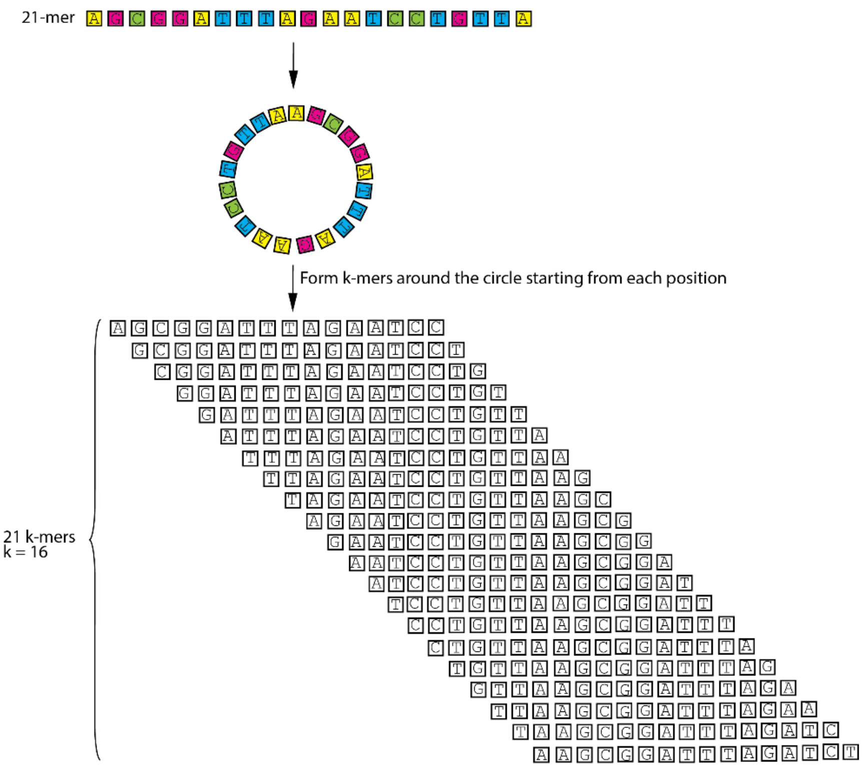

Supplementary Fig. 8: k-mer analysis

Schematic sketch of extracting all 21 possible 16-mers from a 21-mer in a circular k-mer extraction procedure.

Supplementary Note 7: Parameter choice and robustness

The chosen methods of k-mer extraction and Jaccard similarity comprise several parameters that have to be selected before data processing. Namely, *k* has to be selected, and optionally a weighting regimen for the Jaccard coefficient as well as the choice of how many reads to include in the analysis. The latter is designed to exclude the reads with the lowest counts from the analysis and is expressed as a percentage threshold. I.e. a threshold of 0.7 only includes the reads that cumulatively make up 70% of the read count (starting with the highest frequency), and vice versa excludes the 30% lowest-frequency reads. This is an additional measure to remove artefacts and noise while still utilizing the relevant information.

These parameters influence the performance of the algorithm in correctly categorizing the compared sets as belonging to like, or unlike inputs. In order to find well-performing parameters, they were screened by combinatorically applying them to the collected original dataset of 39 individual experiments (refer to Fig. 3(a), (c), and (d)). The screened values were k = [4, 6, 8, 10, 12], weighting = [none, linear, logarithmic] and read frequency threshold = [0.5, 0.7, 0.9]. The optimized metric was the relative distance d of the Jaccard similarity score between the most similar sets resulting from different inputs and the most dissimilar sets resulting from the same input. The parameters achieving the highest distance between the two distributions (same and different inputs) were k = 8, linear weighting and a read frequency threshold of 0.7 (70%). The separation between the like and unlike input distributions is d = 0.256 when applying the selected parameters. The separation only slightly to moderately decreases when changing the values of k and the threshold (Fig. 8a, b). However, the separation is not maintained when switching the weighting regime (Fig. 8c). Reducing (by logarithm) or removing the weighting does not significantly affect the similarity of unlike inputs but leads to a broadening of the like input similarity distribution. This highlights that the read frequency information is highly relevant for response generation, and that the differentiation of similar inputs is significantly derived from changing frequencies rather than the mere presence of different k-mers.

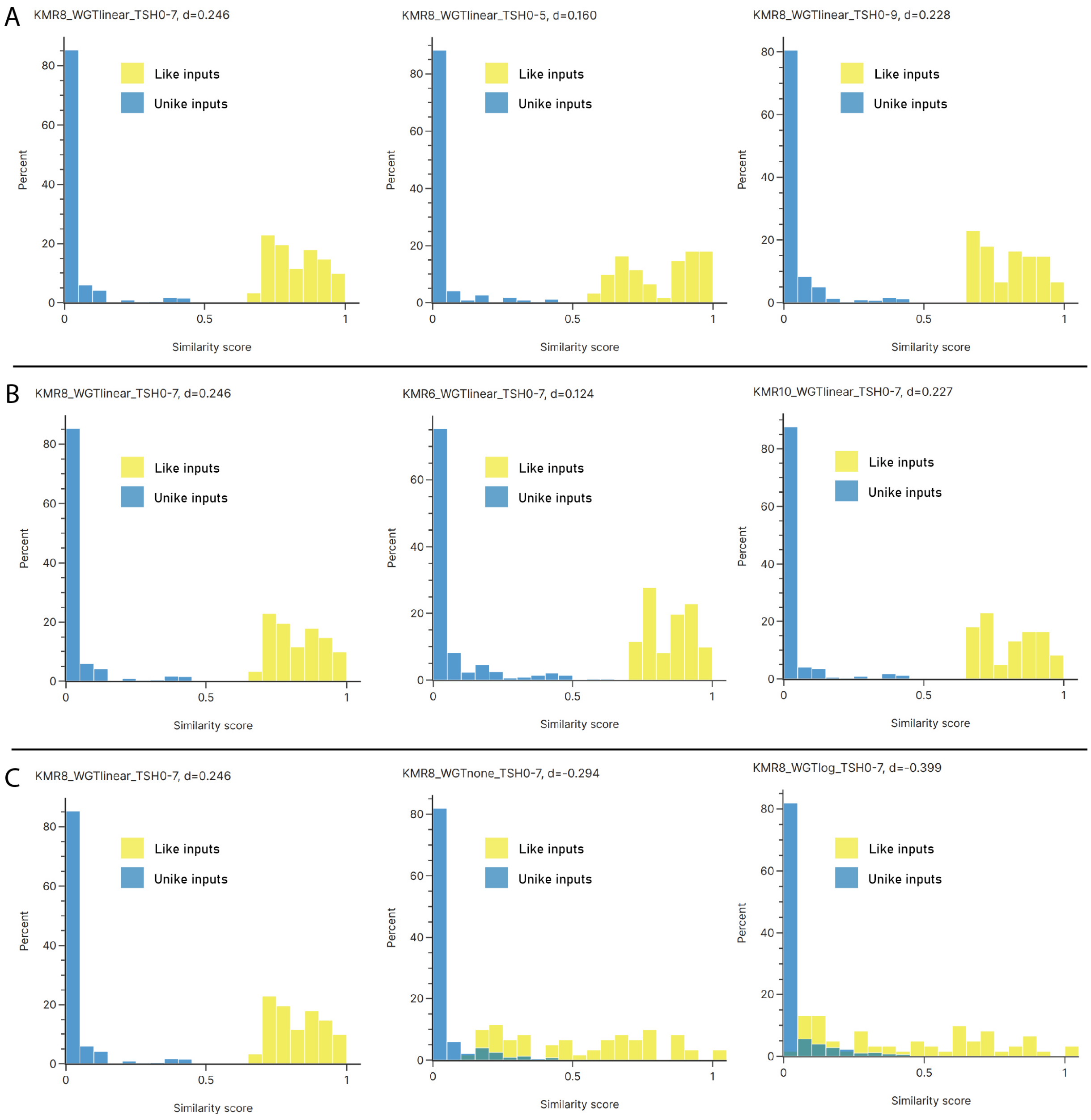

Supplementary Fig. 9: Parameter robustness

Histograms of similarity scores of all like (yellow distribution) and unlike (blue distribution) inputs across experiments 1-39 when using different parameters for k-mer extraction and similarity computation. The parameters and resulting distance d between the two distributions are indicated for each histogram, KMR referring to the value of k, WGT to the weighting applied (linear, logarithmic or none) and TSH to the relative read frequency threshold. A) Histograms showing the effect of varying the read frequency cut at k=8 with linear weighting. B) Histograms showing the effect of varying k while keeping the other parameters constant at TSH=0.7 and WGT=linear. C) Histograms showing the

effect of varying the weighting at constant k=8 and TSH=0.7. Source data are provided as a Source Data file.

Supplementary Note 8: Discussion of the parameters and input constraints

It can be argued that a dataset comprising 39 experiments is not comprehensive and, therefore, that the parameters emerging from the optimization are arbitrary. While this is a valid concern, the dataset on which the parameters were optimized is heavily biased towards sets that are difficult to differentiate. Many of the cross-compared inputs have a minimal Levenshtein distance of 1. Among them were input primers that only differ in their length by a single nucleotide but were otherwise identical, which are expected to draw a highly similar sequence set from the pool in the selection PCR. The chosen parameters perform well in distinguishing these edge cases, showing an error rate of zero. Moreover, they separate the like from the unlike input distribution by a large margin (the minimal distance of Jaccard similarities being d=0.256, and the distance of the means d=0.797). Therefore, these parameters can be expected to perform robustly over all potential inputs.

However, in a use case scenario, it may still be desirable to constrain the allowed inputs to a constant primer length and to limit their GC-content for practical reasons. For example, this would allow running all PCR reactions under the same conditions. When applying the length constraint in accordance with the pool size (as discussed in Supplementary Note 4), the inputs are limited to 13 nucleotides in total, distributed over the two primers. A common recommendation for optimal primer GC-content is approximately 40-60%. Considering that in the current design the input primers contain a constant portion with a fixed GC-content of 50%, the 13 nucleotides thus have to contain at least 3 and max. 10 G/C nucleotides. When applying these constraints to the existing dataset, the poly-T and the variable-length inputs are excluded. The resulting distributions calculated with the previously selected parameters (k = 8, linear weighting and a read frequency threshold of 0.7) are shown in Fig. 9. The distance between the distributions approximately doubles from d=0.256 for the case without constraints to d=0.542 when the length requirement and limited GC content are implemented. Notably, this scenario still includes several inputs differing from each other by a Levenshtein distance of 1. This further highlights the robustness of the chosen method of data processing.

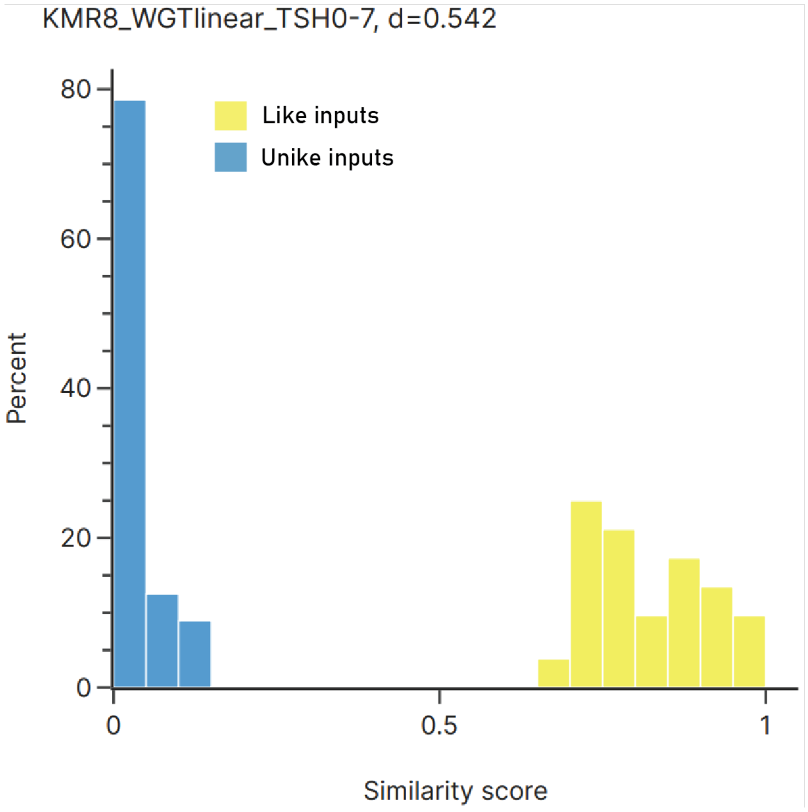

Supplementary Fig. 10: Similarity scores with input constraints

Histograms of similarity scores of all like (yellow distribution) and unlike (blue distribution) inputs across 24 experiments when applying input constraints regarding length and GC-content of the primers and using the parameters k=8, linear weighting and a read frequency threshold of 0.7. Source data are provided as a Source Data file.

Supplementary Note 9: Down-sampling simulation

There are experimental factors that lead to varying read numbers and quality per experiment. To increase efficiency and extrapolate the limits of the system, down-sampling simulations were conducted to find the average minimum number of reads per execution of the CUF that still allows for correct output assignment with high fidelity. The simulation was performed with experiments 1-39 using a python pipeline and comprised stochastically drawing sets of different sizes out of the actual number of reads using the python random number generator and performing feature extraction with the drawn sets. The number of simulations per set size was 30. For samples with less reads than the specified set size the entire read set was included in the analysis. The average distance of separation between similarity scores of like and unlike outputs was then investigated with respect to the drawn set size, the results of which are shown in Fig. 10. A distance of >0 indicates that the similarity distributions of responses stemming from like and unlike inputs are entirely separable, meaning all outputs can be correctly assigned. In contrast, a negative distance implies that certain edge cases would be incorrectly assigned, i.e. two outputs stemming from the same input would be incorrectly assigned as belonging to a different input, or vice versa. From the draws, the probability for one or more response out of the generated dataset being incorrectly assigned was calculated with a one-sided t-test, assuming a normal distribution. The simulations showed that below 10’000 reads, the probability for a distance <0 becomes practically relevant in the case where variable-length inputs are allowed. If length and GC-content constraints are applied (as discussed in Supplementary Note 9), down-sampling indicates that even a read set comprising 1000 sequences can be assigned with a success rate of close to 100%, even with the experiments including inputs with the minimal possible distance from each other. This has a positive impact on affordability, as the sequencing cost associated with 1000 reads is in the range of 10 cents.

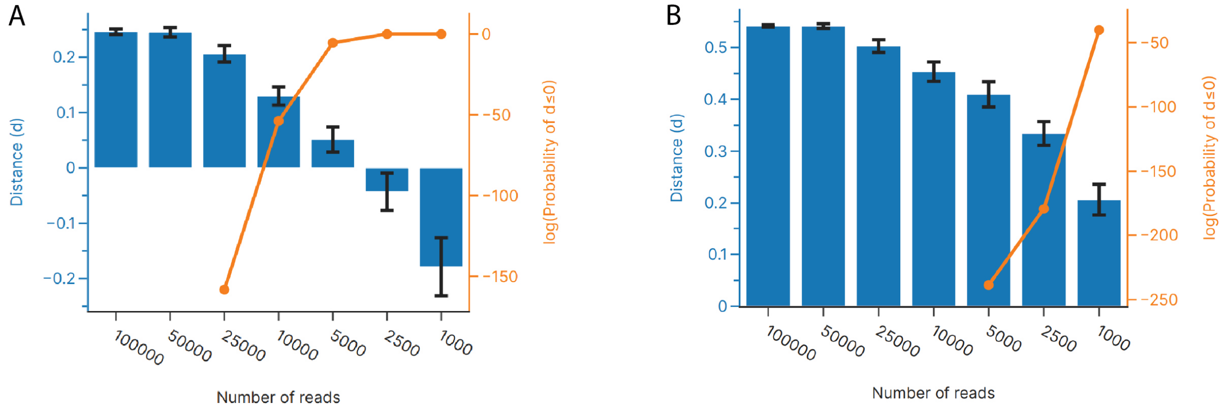

Supplementary Fig. 11*:* Distance (d) after downsampling to the respective number of reads.

The distance refers to the minimal difference in similarity score between the sets resulting from like and unlike inputs after k-mer analysis using the entire dataset with parameters k=8, weight=linear, threshold=0.7. The number of draws per read was n=30. The error bars represent the 95% confidence intervals for the distance resulting from all draws. The curve shows the probability that at a given number of reads the resulting set distance using the indicated parameters will be ≤0, whereby a negative distance means that like and unlike inputs can no longer be completely distinguished. For read numbers lacking a probability indicator the probability exceeded the float limit and thus shows as zero, i.e. is not represented in the log chart. A) Distance d after downsampling to the respective number of reads without applying input constraints. B) Distance d after downsampling to the respective number of reads in the case where the input is constrained with respect to GC-content and length. Source data are provided as a Source Data file.

Supplementary Note 10: Use of MinHash signatures and fuzzy extraction for key generation

A CUF outputs a set S of DNA sequences as a response to an input primer. If the function is evaluated again with the same input primer, it outputs a set of DNA sequences that is similar to the previously generated set S, and if the function is evaluated again with a different input primer, it outputs a set that is different from S. In order to map a response sequence set S to an output of bits (or bytes), we use a MinHash signature[4, 5] followed by a fuzzy vault system[6], which is a variant of a fuzzy extractor[7].

MinHash-based tools are widely used, with numerous applications in genomics, such as taxonomic diversity assessment[8] and metagenome distance estimation[9]. A MinHash maps a set – in this case the k-mers and associated abundances that comprise a response of a CUF – to a signature in form of a vector. The vector dimensions are pre-defined and constant, irrespective of the set size, and the similarity of two compared vectors approximately matches the Jaccard similarity of the respective k-mer sets, from which the signatures were derived.

Specifically, our MinHash function uses a weighted MinHash implementation (datasketch Python library) with the relative abundances of k-mers as weights. This, together with the same selection of k-mer size and threshold, most accurately represents the analysis based on weighted Jaccard similarity of Supplementary Note 7. Each result of the 255 MinHash permutations is converted to one byte by modulo 256, yielding a signature of 255 bytes.

The vectors are then converted to binary keys by the fuzzy vault. This works as follows: Let w be the data from a noisy process - in our setup w is generated from the MinHash signatures obtained from the CUF. A fuzzy vault system generates a random key c, and uses it to generate the helper data h from the data w. The helper data is publicly stored and provides redundancy for error correction but does not give any advantage for reconstructing the original data w or the key c on its own. The original data w is not stored, instead the generated random key c is used for authentication. If new data w' is collected the fuzzy vault system attempts to reconstruct the original key c from the new data w' and the original helper data h. If the new data w' is close (i.e., has a high similarity) to the original data w, then the redundancy included in the helper data h suffices to recover the same key c from w'.

More specifically, based on experiments 1-39, we implemented a fuzzy vault system with a so-called code offset construction[6]. Given the original data w, we generate a 256-bit key c (i.e., 32 bytes) and encode it with a with a Reed-Solomon code (reedsolo Python library, 255-32=223 bytes of

redundancy), which yields a byte string c* of equal length to the MinHash. We then store the helper information h = w – c*, where subtraction is in the finite field the letters of the codeword are in (i.e., bytes in our setup).

At the reconstruction phase, we are given the helper information h and data w', and we compute

$$c' = \ decode(w' - h) = decode(w' - w + c^{*})$$

where decode is the Reed-Solomon decoder. We have that c'= c if the data w' is sufficiently close to the original data w, since then, the redundancy afforded by the Reed-Solomon code allows reconstruction of c = decode(w' – w + c*). Otherwise, reconstruction of c will fail, and no key is generated.

See the code supplement for an implementation of the fuzzy vault system, which correctly reconstructs all code words in the dataset stemming from experiments 1-39 (refer to Fig. 3g).

Supplementary Note 11: Discussion of potential inverse operation and brute force attacks

The response of a CUF to a challenge is the result of the random chemical composition of the orDNA pool. This is a sufficient condition for the CRPs unpredictability. However, for cryptographic security, irreversibility in analogy to a one-way function is needed. This means that a publicly stored output (and the helper data) should not reveal information that can be used to infer the corresponding input. By the CUF's materiality and random design, this is intrinsically the case. This is even further strengthened by the fact that the chemical response – i.e. the sequence reads – is masked by k-mer analysis, MinHashing and Fuzzy extraction, which are computationally infeasible to invert. So not only can the input not be inferred from the output, but it is with current means even infeasible to unambiguously infer the chemical response from the numerical output. This leads to a combination of digital and materially manifested security layers.

However, the possibility of brute force attacks is unavoidable for any one-way function, PUF, or CUF. For our CUF, the most straightforward way to prevent successful guessing of CRPs is to make the pool so large that trial and error to find a given CRP is infeasible within reasonable time and cost. Already at the currently used size of $2.6 \cdot 10^{10}$ sequences, a successful attack to find a CRP by trial and error is unlikely, and CUFs even larger than the ones applied in this work would be conceivable. To read all CRPs with the previously calculated 10'000 reads and at a pool size of $2.6 \cdot 10^{10}$ sequences, one would need ca 260 trillion reads (the equivalent of reading roughly half a million human genomes at a depth of 30). Using state-of-the-art high-throughput sequencing such as the NovaSeq X platform by Illumina, the estimated cost would approach half a billion USD for sequencing alone, not accounting for PCR, time and labor, infrastructure, and other costs.

Additionally, this would require that an adversary malignantly gains physical access to the entire pool in large enough quantities and knows the orDNA's general design. In our practical implementation of the non-copiable CUF tokens, brute force attacks are therefore already prevented by the fact that only a limited number of operations can be performed on a given token.

Supplementary Note 12: Discussion of 2',3'-dideoxy end modification to introduce unclonability

As discussed in the main manuscript, a major constraint for re-creation of a chemical unclonable function is the massive number of sequences, resulting in exorbitant cost of reading and recreating the function. However, should an adversary gain physical access to a given CUF, using the outer handle such an adversary could not only sequence, but also copy the pool using PCR.

Unclonability in the sense of uncopiability is a therefore desirable additional feature in the application of a chemical random function. In order to switch an orDNA pool from the clonable (i.e. copiable) to the unclonable (i.e. uncopiable) state, the constant handles at either end of the sequences are removed via restriction digest. In the chosen implementation, only very short handles of 6 and 7 nucleotides, respectively, remain on both ends. These are too short to function as universal PCR primer binding regions due to their low melting temperature - the optimal T_m of primers being between 55 and 60 ºC with a recommended length of 18-30 nucleotides[10].

To prevent the re-addition of longer handles for PCR amplification, the sticky ends resulting from the restriction digest are subsequently blunted using 2',3'-dideoxy nucleotides. Such dideoxy nucleotides are used in Sanger sequencing[11] and their incorporation results in a 3' end without a free hydroxy group. This means no ligation can performed, as the 3'-hydroxy group is necessary for the formation of a phosphodiester bond. The incorporation of dideoxy nucleotides is stable, i.e. cannot be efficiently removed.[12]

To demonstrate that ligation is no longer possible, a CUF comprising approx. 10^8 unique sequences treated with PleI and blunted with dideoxy nucleotides was sent to FASTERIS SA, an external service provider, where it underwent a standard ligation procedure for genomic library preparation. The selected ligation protocol is thus optimized for wide application and performs well in adding adapter sequences to diverse libraries. Two controls were treated along with the test sample, one consisting of the untreated orDNA library, the second one being digested and blunted, but using regular dNTPs instead of dideoxy analogs. The total amount of DNA provided to the service provider was approx. 200 ng for all three samples, which is several orders of magnitude more than would be used for a PCR reaction.

The ligation results and quality control data are shown in Supplementary **Fig.** . S11. While both control libraries were successfully ligated with high yields, the dideoxy-treated CUF yielded no significant product amounts of the expected length, as evidenced by the gel and the electropherogram. This indicates that dideoxy blunting is indeed a suitable measure to make cloning of the function impossible, or at least extremely difficult. Any intruder who attempts to copy such a CUF would not only have to get physical access to an unrealistically high amount of the original function, but is likely to introduce bias and change the structure of the orDNA in a way that would be evident and impair its functionality.

The modification additionally introduces obstacles to simultaneous sequencing of the entire pool, as this typically involves library preparation by adding adapters, either by PCR or by ligation. This also includes “PCR-free” methods, such as nanopore sequencing. The truncated and modified 3’-ends would need to be circumvented, which, even if possible, would increase the complexity and cost of sequencing and copying further.

In conclusion, it can be shown that, aside from the necessity of gaining access to the pool with malicious intent, there is no straightforward way to read and copy the pool. Any such attempt would increase the effort, cost and complexity, and introduce the risk of altering the pool in a way that hampers its performance as a random function.

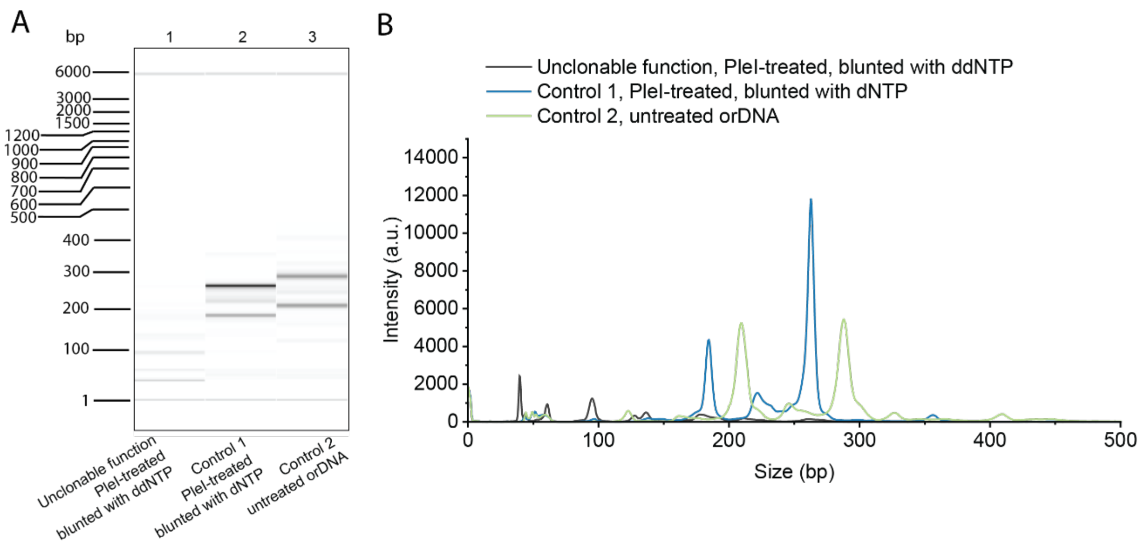

Supplementary Fig. 12: Ligation results

A) gel image and B) electropherogram, as received from FASTERIS SA. The expected length of the fully ligated fragments is 263 bp for samples 1 and 2, and 289 bp for sample 3. The fragments araund

185 and 210 bp correspond to the one-ended ligation. The labels were added to the gel image by the authors. The electropherograms were plotted from the raw data provided by FASTERIS SA. Source data are provided as a Source Data file.

Supplementary Note 13: Cost estimation

The cost of a CUF is composed of the cost for the function itself and of its operation. For this study, 0.5 exabyte (4g of an orDNA library) were commercially ordered for a price of ca 90 USD. This corresponds to less than 20 cents per 1000 Terabyte of entropy in the form of operable random DNA. This amount of orDNA in theory suffices for billions of individual CUFs as implemented in this study, meaning the cost of random synthesis is insignificant in any realistic scenario at scale. However, generating a CUF from orDNA requires a PCR reaction to generate multiple copies, followed by chemical modifications to switch the function from the clonable to the unclonable state. Within the scales applied in this study, the costs for these steps approximately follow a linear relationship with regards to the function size. For a CUF comprising 10^8 unique sequences, the singleplex qPCR using an intercalating dye in 20 µl reaction volume with standard primers costs ca 50 cents in reagents and consumables at scale. The material generated by a single PCR reaction was on average sufficient for ca. 200 executions of the CUF, but this is scalable.

The restriction digest with PleI costs ca 1 USD/assay in reagents and consumables, again for the amount needed for 200 executions of the function. End blunting with ddNTPs costs ca. 3 USD; although this calculation takes a prudent approach with the ddNTPs and enzyme added in large excess. It is therefore likely that further process optimization would cut the cost significantly.

Adding up all cost items for function generation – from DNA synthesis to generating the double-stranded orDNA to switching it to the unclonable state – results in a price of around 4.6 USD per CUF.

At least wo executions are performed per input (one for registration, one or more for authentication events). For a single function execution, 4 consecutive PCR reactions, including adding the sequencing adapters, are performed, followed by Illumina sequencing. The cost for one operation from input to output is comprised of reagents and consumables for PCR and next generation sequencing (see Supplementary Table 1). For the sequencing cost, it was assumed that 10’000 reads are needed for a single execution, which is a prudent estimate based on the down-sampling experiments assuming no input constraints (as discussed in Supplementary Note 9). The current price of an iSeq 100 reagent kit was taken as a benchmark and broken down to the defined number of required reads.

In summary, the cost of generating the function lies below 1% and is thus insignificant. The combined cost is instead dominated by the price for sequencing and PCR reagents, which currently make up

80% of the entire amount per function execution. It is important to note that the assumptions underlying the sequencing cost estimate are deliberately conservative and based on the current procedure; when reducing the required reads to 1000 and assuming the use of a large-scale sequencing platform, the sequencing costs would drop by approx. two orders of magnitude. In that case, the costs for PCR reagents and consumables would dominate the overall price at approx. 2 USD.

A caveat of the above calculations is that most of the items do not involve labor, as this is hard to estimate in the setting of a research laboratory. Moreover, an assessment of the current labor requirement would not be a realistic benchmark, as most of the operations are of low complexity and would be automatized in a large-scale setting. The remaining hands-on work can easily be parallelized in large multi-well plates, processing many function operations at the same time. Thus, at scale, labor is not expected to have a large impact on the overall cost.

Supplementary Table 1

Approximated item-by-item cost compilation for generation of a function comprising ca 10^8 sequences.

**Step**	**Cost item**	**Approximated cost at scale (USD)**	**Cost per execution***
Synthesis	Library	<0.01	<0.0001
orDNA generation	Mastermix (e.g. KAPA SYBR FAST, KAPA Biosystems, Wilmington, USA)	0.35	<0.01
	PCR primers	<0.01	<0.0001
	Consumables	0.15	0.0015
	Other reagents	0.10	0.001
End modification	Restriction enzyme	0.80	0.008
	Consumables	0.20	0.002
	Sequenase (e.g. ThermoFisher)	1.30	0.0013
	ddNTP mix	1.80	0.0018
	Other reagents	0.10	0.001
**Total**		**4.80**	**0.048**

*calculated based on the assumption of 200 executions per function

Supplementary Table 2

Approximated item-by-item cost compilation for a single execution of a function comprising ca 10^8 sequences.

**Step**	**Cost item**	**Approximated cost per function execution**
**Function operation**	Mastermix (e.g. KAPA SYBR FAST, KAPA Biosystems, Wilmington, USA)	1.40
	PCR primers	0.05
	Consumables	0.60
	Illumina sequencing (iSeq 100 reagent kit)	1.50
	Other reagents	0.10
**Total**		**3.65**

Supplementary Table 3: Sequence and primer list

Name	Sequence 5'-3'
Library 1	ATGCGATGCAGTAAGCACTCNNNNNNNNNACACGACGCTCTTCCGATCTN NNNNNNNNNNNNNNNNNNNNGCTCAGGATACCAAGCTGTCCNNNNNNNN NNGATATCTGCTCGGACCGCTA
Library 2	ATGCGAGTCAGATNGCACTCNNNNNNNNNACACGACGCTCTTCCGATCTN NNNNNNNNNNNNNNNNNNNNGCTCAGGATACCAAGCTGTCCNNNNNNNN NNGACATNGGACGACTCAGCTA
Library 1 Handle primer fw	ATGCGATGCAGTAAGCACTC
Library 1 Handle primer rv	TAGCGGTCCGAGCAGATATC
Library 2 Handle primer fw	ATGCGAGTCAGATNGCACTC
Library 2 Handle primer rv	TAGCTGAGTCGTCCNATGTC
Input primer infw1	AGT AAG CAC TCG CTT ACG AC
Input primer inrv1	GAG CAG ATA TCA TTG GCA ACG
Input primer infw4	TGCAGTAAGCACTCTACGAC
Input primer inrv5	TCCGAGCAGATATCGGCAACG
Input primer infw4.1	TGCAGTAAGCACTCTACGAT
Input primer inrv5.2	TCCGAGCAGATATCAGCAACG
Input primer infw4.3	TGCAGTAAGCACTCAGGTCG
Input primer inrv5.3	TCCGAGCAGATATCATTCTTC
Input primer infw4.4	TGC AGT AAG CAC TCT TTT TT
Input primer inrv5.4	TCC GAG CAG ATA TCT TTT TTT
Input primer infw5	GCAGTAAGCACTCTTACGAC
Input primer inrv6	CCGAGCAGATATCTGGCAACG
Input primer infw6	CAGTAAGCACTCGTTACGAC

Input primer inrv7	CGAGCAGATATCTTGGCAACG
Input primer inrv8	GTCCGAGCAGATATCGCAACG
Input primer infw9	ATGCAGTAAGCACTCTACGA
Input primer inrv9	ATGCAGTAAGCACTCTACGA
Trimming primer fw	ACACGACGCTCTTCCGATCT
Trimming primer rv	GGACAGCTTGGTATCCTGAGC
Illumina primer 1F	ACACTCTTTCCCTACACGACGCTCTTCCGATCT
Illumina primer 1R-AL	GTGACTGGAGTTCAGACGTGTGCTCTTCCGATCTGGACAGCTTGGTATCCTGAGC
Illumina primer 2FU	AATGATACGGCGACCACCGAGATCTACACTCTTTCCCTACACGACGC
Illumina primer 2RI Index 1	CAAGCAGAAGACGGCATACGAGATCGTGATGTGACTGGAGTTCAGACGTGT
Illumina primer 2RI Index 2	CAAGCAGAAGACGGCATACGAGATACATCGGTGACTGGAGTTCAGACGTGT
Illumina primer 2RI Index 3	CAAGCAGAAGACGGCATACGAGATGCCTAAGTGACTGGAGTTCAGACGTGT
Illumina primer 2RI Index 4	CAAGCAGAAGACGGCATACGAGATTGGTCAGTGACTGGAGTTCAGACGTGT
Illumina primer 2RI Index 5	CAAGCAGAAGACGGCATACGAGATCACTGTGTGACTGGAGTTCAGACGTGT
Illumina primer 2RI Index 6	CAAGCAGAAGACGGCATACGAGATATTGGCGTGACTGGAGTTCAGACGTGT
Illumina primer 2RI Index 7	CAAGCAGAAGACGGCATACGAGATGATCTGGTGACTGGAGTTCAGACGTGT
Illumina primer 2RI Index 8	CAAGCAGAAGACGGCATACGAGATTCAAGTGTGACTGGAGTTCAGACGTGT
Illumina primer 2RI Index 9	CAAGCAGAAGACGGCATACGAGATCTGATCGTGACTGGAGTTCAGACGTGT
Illumina primer 2RI Index 10	CAAGCAGAAGACGGCATACGAGATAAGCTAGTGACTGGAGTTCAGACGTGT

Illumina primer 2RI Index 11	CAAGCAGAAGACGGCATACGAGATGTAGCCGTGACTGGAGTTCAGACGTGT
Illumina primer 2RI Index 12	CAAGCAGAAGACGGCATACGAGATTACAAGGTGACTGGAGTTCAGACGTGT
Illumina primer 2RI Index 13	CAAGCAGAAGACGGCATACGAGATTTGACTGTGACTGGAGTTCAGACGTGT
Illumina primer 2RI Index 14	CAAGCAGAAGACGGCATACGAGATGGAACTGTGACTGGAGTTCAGACGTGT
Illumina primer 2RI Index 15	CAAGCAGAAGACGGCATACGAGATTGACATGTGACTGGAGTTCAGACGTGT
Illumina primer 2RI Index 16	CAAGCAGAAGACGGCATACGAGATGGACGGGTGACTGGAGTTCAGACGTGT
Illumina primer 2RI Index 17	CAAGCAGAAGACGGCATACGAGATCTCTACGTGACTGGAGTTCAGACGTGT

Supplementary Table 4: List of experiments

Experi-ment number	Experi-ment desig-nation	CUF description	Input primers	Input bases	Sequencing file name
1	1	CUF S1, 10^8 sequences	infw4/inrv5	TACGAC / GGCAACG	Sp1-Infw4-Inrv5-1_S5_L001_R1_001.fastq
2	2	CUF S1, 10^8 sequences	infw4/inrv5	TACGAC/ GGCAACG	Sp1-Infw4-Inrv5-2_S6_L001_R1_001.fastq
3	3	CUF S1, 10^8 sequences	infw4/inrv5	TACGAC/ GGCAACG	Infw4-Inrv5-1_S1_L001_R1_001.fastq
4	4	CUF S1, 10^8 sequences	infw4/inrv5	TACGAC/ GGCAACG	Infw4-Inrv5-2_S2_L001_R1_001.fastq
5	P1.1	CUF S1 , Proliferation 1, 10^8 sequences	infw4/inrv5	TACGAC/ GGCAACG	Sp1-amp-Infw4-Inrv5-1_S11_L001_R1_001.fastq
6	P1.2	CUF S1 , Proliferation 1, 10^8 sequences	infw4/inrv5	TACGAC/ GGCAACG	Sp1-amp-Infw4-Inrv5-2_S12_L001_R1_001.fastq
7	P2	CUF S1 , Proliferation 2, 10^8 sequences	infw4/inrv5	TACGAC/ GGCAACG	0_S1_L001_R1_001.fastq
8	P3	CUF S1 , Proliferation 3, 10^8 sequences	infw4/inrv5	TACGAC/ GGCAACG	1_S2_L001_R1_001.fastq
9	P4	CUF S1 , Proliferation 4, 10^8 sequences	infw4/inrv5	TACGAC/ GGCAACG	2_S3_L001_R1_001.fastq

10	P5	CUF S1 , Proliferation 5, 10^8 sequences	infw4/inrv5	TACGAC/ GGCAACG	3_S4_L001_R1_001.fastq
11	5	CUF S1, 10^8 sequences	infw4.1/inrv5	TACGAT/ GGCAACG	Sp1-Infw4-1-Inrv5-1_S9_L001_R1_001.fastq
12	6	CUF S1, 10^8 sequences	infw4.1/inrv5	TACGAT/ GGCAACG	Sp1-Infw4-1-Inrv5-2_S10_L001_R1_001.fastq
13	7	CUF S1, 10^8 sequences	infw4/inrv5.2	TACGAC/ AGCAACG	Sp1-Infw4-Inrv5-2-1_S11_L001_R1_001.fastq
14	8	CUF S1, 10^8 sequences	infw4/inrv5.2	TACGAC/ AGCAACG	Sp1-Infw4-Inrv5-2-2_S12_L001_R1_001.fastq
15	9	CUF S1, 10^8 sequences	infw4.3/inrv5.3	AGGTCG/ AATCATG	Sp1-Infw4-3-Inrv5-3-2_S14_L001_R1_001.fastq
16	10	CUF S1, 10^8 sequences	infw4.3/inrv5.3	AGGTCG/ AATCATG	Sp1-Infw4-3-Inrv5-3-2_S14_L001_R1_001.fastq
17	11	CUF S1, 10^8 sequences	infw4.4/inrv5.4	TTTTTT/ TTTTTTT	Lib4-Sp1-G2-infw4-4-inrv5-4-1_S13_L001_R1_001.fastq
18	12	CUF S1, 10^8 sequences	infw4.4/inrv5.4	TTTTTT/ TTTTTTT	Lib4-Sp1-G2-infw4-4-inrv5-4-2_S14_L001_R1_001.fastq
19	13	CUF S1, 10^8 sequences	infw4/inrv8	TACGAC/ GCAACG	Sp1-Infw4-Inrv8-1_S9_L001_R1_001.fastq
20	14	CUF S1, 10^8 sequences	infw4/inrv8	TACGAC / GCAACG	Sp1-Infw4-Inrv8-2_S10_L001_R1_001.fastq
21	15	CUF S1, 10^8 sequences	infw9/inrv9	TACGA / GGCAAC	8_S9_L001_R1_001.fastq
22	16	CUF S1, 10^8 sequences	infw9/inrv9	TACGA / GGCAAC	9_S10_L001_R1_001.fastq

23	17	CUF S1, 10^8 sequences	infw1/inrv1	GCTTACGAC / ATTGGCAACG	Infw1-Inrv1-2_S3_L001_R1_001.fastq
24	18	CUF S1, 10^8 sequences	infw1/inrv1	GCTTACGAC / ATTGGCAACG	Sp1-Infw1-Inrv1-1_S7_L001_R1_001.fastq
25	19	CUF S1, 10^8 sequences	infw1/inrv1	GCTTACGAC / ATTGGCAACG	Sp1-Infw1-Inrv1-2_S8_L001_R1_001.fastq
26	20	CUF S2, 10^8 sequences	infw4/inrv5	TACGAC / GGCAACG	Sp2-Infw4-Inrv5-1_S1_L001_R1_001.fastq
27	21	CUF S2, 10^8 sequences	infw4/inrv5	TACGAC / GGCAACG	Sp2-Infw4-Inrv5-2_S2_L001_R1_001.fastq
28	22	CUF S2, 10^8 sequences	infw1/inrv1	GCTTACGAC / ATTGGCAACG	Sp2-Infw1-Inrv1-1_S3_L001_R1_001.fastq
29	23	CUF S2, 10^8 sequences	infw1/inrv1	GCTTACGAC / ATTGGCAACG	Sp2-Infw1-Inrv1-2_S4_L001_R1_001.fastq
30	24	CUF S3, 10^8 sequences	infw4/inrv5	TACGAC / GGCAACG	Sp3-Infw4-Inrv5-1_S3_L001_R1_001.fastq
31	25	CUF S3, 10^8 sequences	infw4/inrv5	TACGAC / GGCAACG	Sp3-Infw4-Inrv5-2_S4_L001_R1_001.fastq
32	26	CUF M1, 1.6 x 10^9 sequences	infw5/inrv6	TTACGAC / TGGCAACG	Sp-e9-Inrv5-Inrv6-1_S5_L001_R1_001.fastq

33	27	CUF M1, 1.6 x 10^9 sequences	infw5/inrv6	TTACGAC / TGGCAACG	Sp-e9-Inrv5-Inrv6-2_S6_L001_R1_001.fastq
34	28	CUF M1, 1.6 x 10^9 sequences	infw5/inrv5	TTACGAC / GGCAACG	Sp-e9-Inrv5-Inrv5-1_S7_L001_R1_001.fastq
35	29	CUF M1, 1.6 x 10^9 sequences	infw5/inrv5	TTACGAC / GGCAACG	Sp-e9-Inrv5-Inrv5-2_S8_L001_R1_001.fastq
36	30	CUF L1, 2.6 x 10^10 sequences	infw6/inrv7	GTTACGAC / TTGGCAACG	Sp-e10-Infw6-Inrv7-1_S15_L001_R1_001.fastq
37	31	CUF L1, 2.6 x 10^10 sequences	infw6/inrv7	GTTACGAC / TTGGCAACG	Sp-e10-Infw6-Inrv7-2_S16_L001_R1_001.fastq
38	32	CUF L1, 2.6 x 10^10 sequences	infw6/inrv6	GTTACGAC / TGGCAACG	Sp-e10-Infw6-Inrv6-1_S13_L001_R1_001.fastq
39	33	CUF L1, 2.6 x 10^10 sequences	infw6/inrv6	GTTACGAC / TGGCAACG	Sp-e10-Infw6-Inrv6-2_S14_L001_R1_001.fastq
40	C2 P1*	CUF S1 , Proliferation 1, 10^8 sequences	infw4.1/inrv5	TACGAT/ GGCAACG	P1-infw4-1inrv5_S4_L001_R1_001.fastq
41	C2 P2*	CUF S1 , Proliferation 2, 10^8 sequences	infw4.1/inrv5	TACGAT/ GGCAACG	P1-infw4-1inrv5_S5_L001_R1_001.fastq
42	C2 P3*	CUF S1 , Proliferation 3, 10^8 sequences	infw4.1/inrv5	TACGAT/ GGCAACG	P1-infw4-1inrv5_S6_L001_R1_001.fastq
43	C2 P4*	CUF S1 , Proliferation 4, 10^8 sequences	infw4.1/inrv5	TACGAT/ GGCAACG	P1-infw4-1inrv5_S7_L001_R1_001.fastq

44	C2 P5*	CUF S1 , Proliferation 5, 10^8 sequences	infw4.1/inrv5	TACGAT/ GGCAACG	P1-infw4-1inrv5_S8_L001_R1_001.fastq
45	C4 P1*	CUF S1 , Proliferation 1, 10^8 sequences	infw4.3/inrv5.3	AGGTCG/ AATCATG	P1-infw4-3inrv5-3_S14_L001_R1_001.fastq
46	C4 P2*	CUF S1 , Proliferation 2, 10^8 sequences	infw4.3/inrv5.3	AGGTCG/ AATCATG	P1-infw4-3inrv5-3_S15_L001_R1_001.fastq
47	C4 P3*	CUF S1 , Proliferation 3, 10^8 sequences	infw4.3/inrv5.3	AGGTCG/ AATCATG	P1-infw4-3inrv5-3_S16_L001_R1_001.fastq
48	C4 P4*	CUF S1 , Proliferation 4, 10^8 sequences	infw4.3/inrv5.3	AGGTCG/ AATCATG	P1-infw4-3inrv5-3_S17_L001_R1_001.fastq
49	C4 P5*	CUF S1 , Proliferation 5, 10^8 sequences	infw4.3/inrv5.3	AGGTCG/ AATCATG	P1-infw4-3inrv5-3_S18_L001_R1_001.fastq
50	C3 P1*	CUF S1 , Proliferation 1, 10^8 sequences	infw4/inrv5.2	TACGAC/ AGCAACG	P1-infw4inrv5-2_S9_L001_R1_001.fastq
51	C3 P2*	CUF S1 , Proliferation 2, 10^8 sequences	infw4/inrv5.2	TACGAC/ AGCAACG	P1-infw4inrv5-2_S10_L001_R1_001.fastq
52	C3 P3*	CUF S1 , Proliferation 3, 10^8 sequences	infw4/inrv5.2	TACGAC/ AGCAACG	P1-infw4inrv5-2_S11_L001_R1_001.fastq
53	C3 P4*	CUF S1 , Proliferation 4, 10^8 sequences	infw4/inrv5.2	TACGAC/ AGCAACG	P1-infw4inrv5-2_S12_L001_R1_001.fastq

54	C3 P5*	CUF S1 , Proliferation 5, 10^8 sequences	infw4/inrv5.2	TACGAC/ AGCAACG	P1-infw4inrv5-2_S13_L001_R1_001.fastq

498 Experiment designation corresponds to the nomenclature used in the main manuscript.

Supplementary Table 5: List of Selection PCR parameters and results

Experiment number	Experi-ment designation	Input primers	Input bases	Selection PCR cycles	Ct	Annealing temp. (Ta)
1	1	infw4/inrv5	TACGAC / GGC AACG	29	17.54	62
2	2	infw4/inrv5	TACGAC / GGCAACG	29	17.54	62
3	3	infw4/inrv5	TACGAC / GGCAACG	29	17.43	62
4	4	infw4/inrv5	TACGAC / GGCAACG	29	17.51	62
5	P1.1	infw4/inrv5	TACGAC / GGCAACG	30	14.4	62
6	P1.2	infw4/inrv5	TACGAC / GGCAACG	30	14.08	62
7	P2	infw4/inrv5	TACGAC / GGCAACG	25	16.25	62
8	P3	infw4/inrv5	TACGAC / GGCAACG	**25**	16.49	62
9	P4	infw4/inrv5	TACGAC / GGCAACG	25	16.68	62
10	P5	infw4/inrv5	TACGAC / GGCAACG	25	15.72	62
11	5	infw4.1/inrv5	TACGAT / GGCAACG	50	21.12	62
12	6	infw4.1/inrv5	TACGAT / GGCAACG	50	21.89	62
13	7	infw4/inrv5.2	TACGAC / AGCAACG	50	20.04	62

14	8	infw4/inrv5.2	TACGAC / AGCAACG	50	19.78	62
15	9	infw4.3/inrv5.3	AGGTCG / AATCATG	50	21.88	62
16	10	infw4.3/inrv5.3	AGGTCG / AATCATG	50	20.86	62
17	11	infw4.4/inrv5.4	TTTTTT / TTTTTTT	36	19.78	56
18	12	infw4.4/inrv5.4	TTTTTT / TTTTTTT	36	19.65	56
19	13	infw4/inrv8	TACGAC / GCAACG	25	15.5	62
20	14	infw4/inrv8	TACGAC / GCAACG	25	15.59	62
21	15	infw9/inrv9	TACGA / GGCAAC	24	11.9	62
22	16	infw9/inrv9	TACGA / GGCAAC	24	12.13	62
23	17	infw1/inrv1	GCTTACGAC / ATTGGCAACG	45	39.43	62
24	18	infw1/inrv1	GCTTACGAC / ATTGGCAACG	50	41.4	62
25	19	infw1/inrv1	GCTTACGAC / ATTGGCAACG	50	39.71	62
26	20	infw4/inrv5	TACGAC / GGCAACG	30	17.27	62
27	21	infw4/inrv5	TACGAC / GGCAACG	30	17.2	62
28	22	infw1/inrv1	GCTTACGAC / ATTGGCAACG	50	35.71	62

29	23	infw1/inrv1	GCTTACGAC / ATTGGCAACG	50	35.61	62
30	24	infw4/inrv5	TACGAC / GGCAACG	25	16.31	62
31	25	infw4/inrv5	TACGAC / GGCAACG	25	16.82	62
32	26	infw5/inrv6	TTACGAC / TGGCAACG	25	17.23	62
33	27	infw5/inrv6	TTACGAC / TGGCAACG	25	18.15	62
34	28	infw5/inrv5	TTACGAC / GGCAACG	25	17.35	62
35	29	infw5/inrv5	TTACGAC / GGCAACG	25	17.93	62
36	30	infw6/inrv7	GTTACGAC / TTGGCAACG	25	19.9	62
37	31	infw6/inrv7	GTTACGAC / TTGGCAACG	25	19.99	62
38	32	infw6/inrv6	GTTACGAC / TGGCAACG	25	18.69	62
39	33	infw6/inrv6	GTTACGAC / TGGCAACG	25	18.53	62
40	C2 P1*	infw4.1/inrv5	TACGAT/ GGCAACG	30	17.27	62
41	C2 P2*	infw4.1/inrv5	TACGAT/ GGCAACG	30	17.01	62
42	C2 P3*	infw4.1/inrv5	TACGAT/ GGCAACG	30	18.19	62

43	C2 P4*	infw4.1/inrv5	TACGAT/GGCAACG	30	18.12	62
44	C2 P5*	infw4.1/inrv5	TACGAT/GGCAACG	30	19.14	62
45	C4 P1*	infw4.3/inrv5.3	AGGTCG/AATCATG	30	19.65	62
46	C4 P2*	infw4.3/inrv5.3	AGGTCG/AATCATG	30	19.47	62
47	C4 P3*	infw4.3/inrv5.3	AGGTCG/AATCATG	30	19.97	62
48	C4 P4*	infw4.3/inrv5.3	AGGTCG/AATCATG	30	18.87	62
49	C4 P5*	infw4.3/inrv5.3	AGGTCG/AATCATG	30	19.47	62
50	C3 P1*	infw4/inrv5.2	TACGAC/AGCAACG	30	19.70	62
51	C3 P2*	infw4/inrv5.2	TACGAC/AGCAACG	30	20.32	62
52	C3 P3*	infw4/inrv5.2	TACGAC/AGCAACG	30	20.53	62
53	C3 P4*	infw4/inrv5.2	TACGAC/AGCAACG	30	20.72	62
54	C3 P5*	infw4/inrv5.2	TACGAC/AGCAACG	30	20.58	62

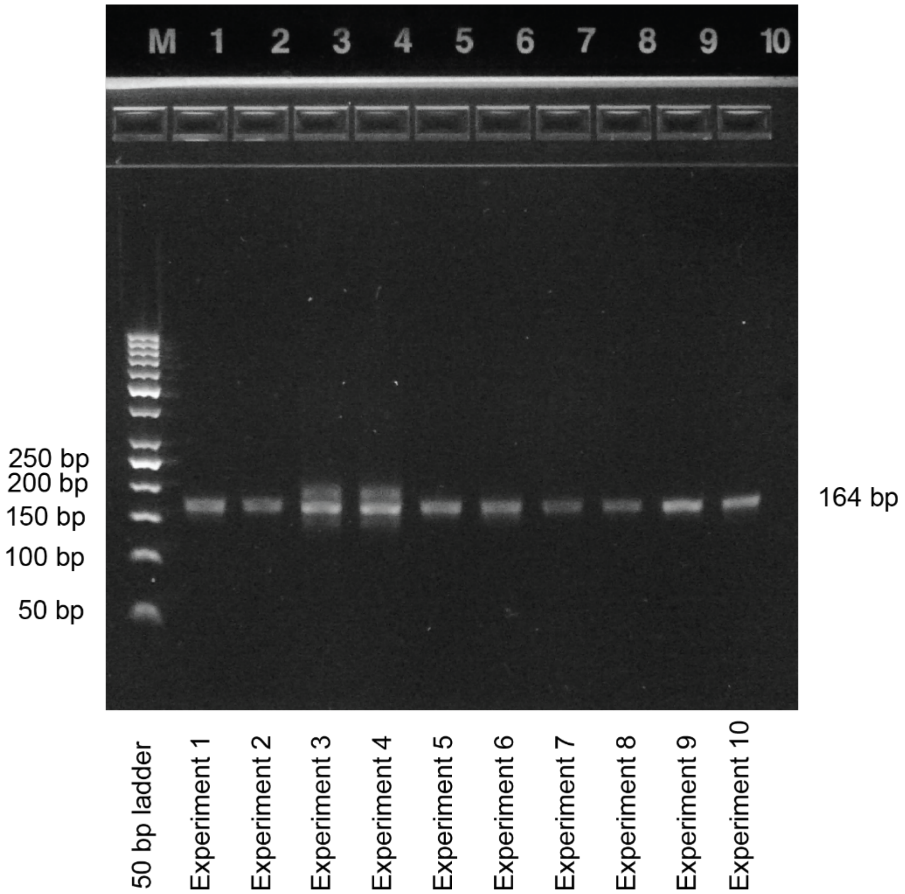

Supplementary Fig. 13

Agarose gel electrophoresis of experiments 1-10 (purified samples for sequencing). The image was converted to grayscale and adjusted for brightness and contrast.

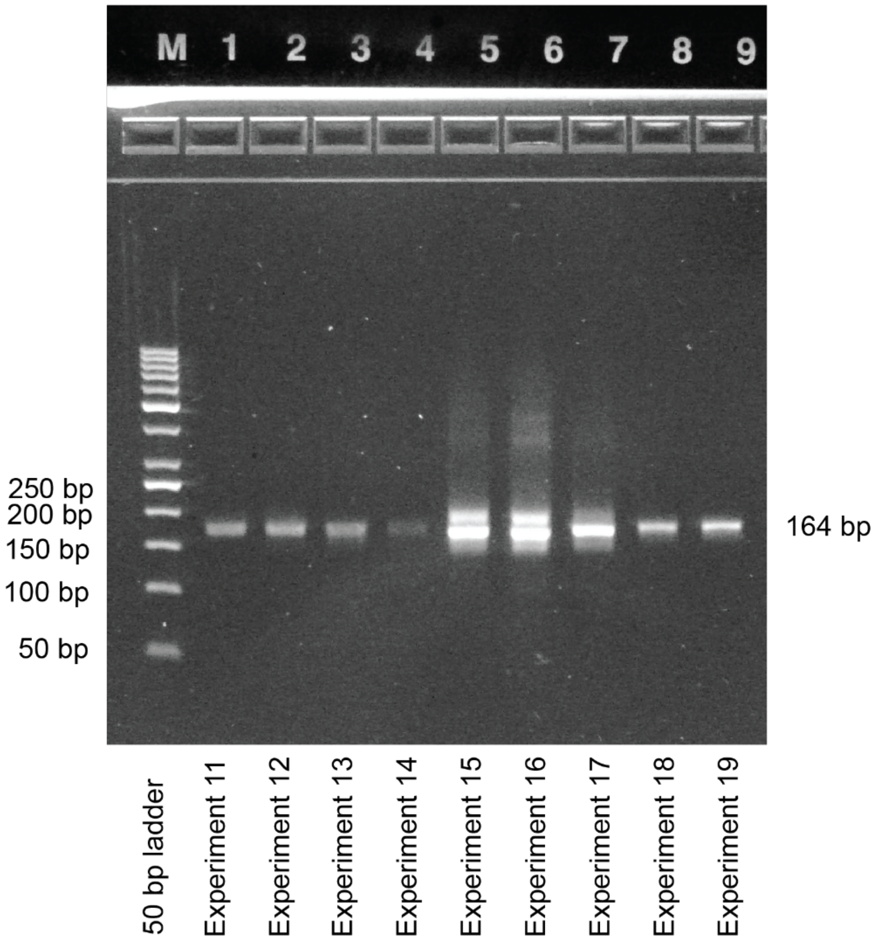

Supplementary Fig. 14

Agarose gel electrophoresis experiments 11-19 (purified samples for sequencing). The image was converted to grayscale and adjusted for brightness and contrast.

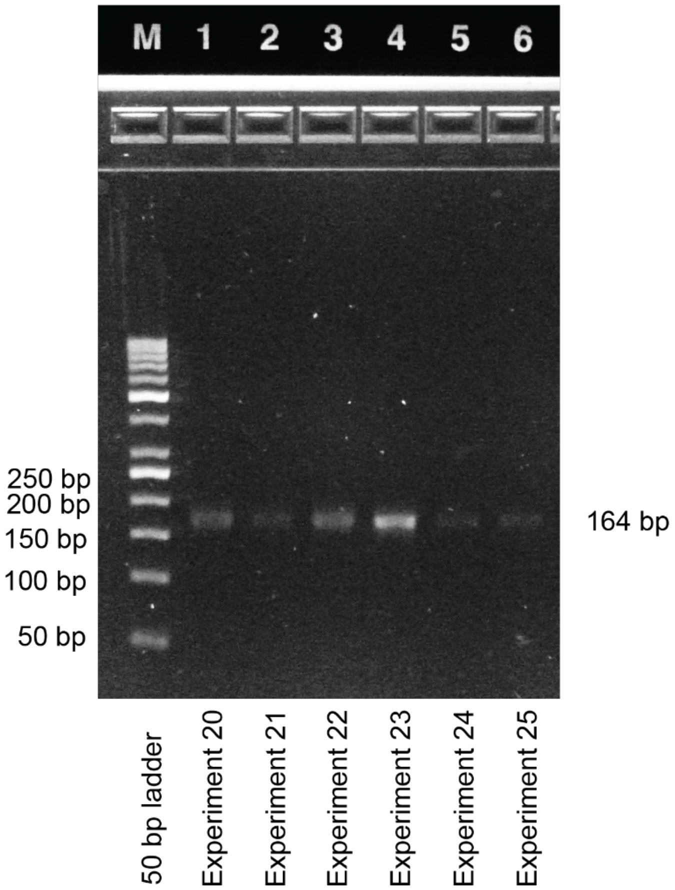

Supplementary Fig. 15

Agarose gel electrophoresis experiments 20-25 (purified samples for sequencing). The image was converted to grayscale and adjusted for brightness and contrast.

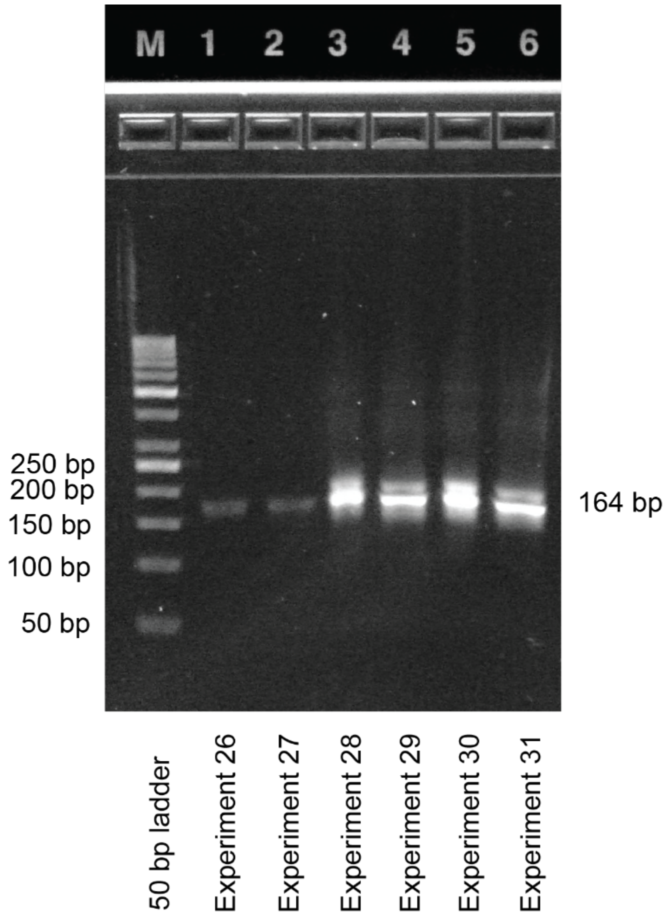

Supplementary Fig. 16

Agarose gel electrophoresis experiments 26-31 (purified samples for sequencing). The image was converted to grayscale and adjusted for brightness and contrast.

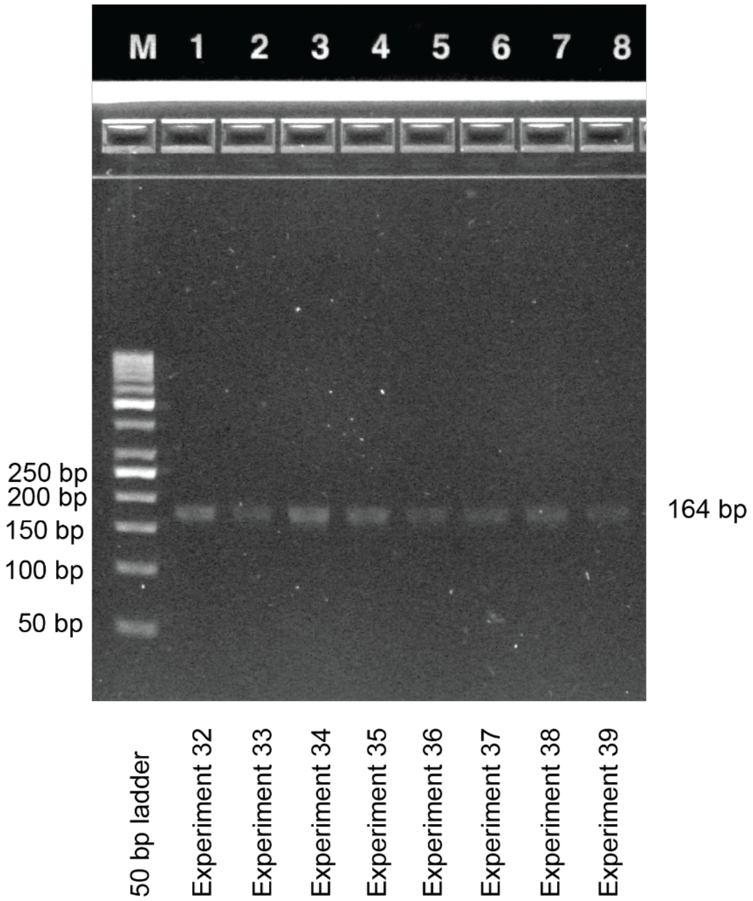

Supplementary Fig. 4

Agarose gel electrophoresis experiments 32-39 (purified samples for sequencing). The image was converted to grayscale and adjusted for brightness and contrast.

References

1. Meiser L. C., Koch J., Antkowiak P. L., Stark W. J., Heckel R.& Grass R. N. DNA synthesis for true random number generation. *Nat. Commun.* **11**, 1-9 (2020).

2. Liu S.& Koslicki D. CMash: fast, multi-resolution estimation of k-mer-based Jaccard and containment indices. *Bioinformatics* **38**, i28-i35 (2022).

3. Besta M.*, et al.* Communication-efficient jaccard similarity for high-performance distributed genome comparisons. In: *2020 IEEE International Parallel and Distributed Processing Symposium (IPDPS)*). IEEE (2020).

4. Broder A. Z. On the resemblance and containment of documents. In: *Proceedings. Compression and Complexity of SEQUENCES 1997 (Cat. No. 97TB100171)*). IEEE (1997).

5. Haveliwala T. H., Gionis A.& Indyk P. Scalable Techniques for Clustering the Web (extended Abstract). In: *WebDB (informal proceedings)*). Citeseer (2000).

6. Juels A.& Sudan M. A fuzzy vault scheme. *Designs, Codes and Cryptography* **38**, 237-257 (2006).

7. Dodis Y., Ostrovsky R., Reyzin L.& Smith A. Fuzzy Extractors: How to Generate Strong Keys from Biometrics and Other Noisy Data. *SIAM Journal on Computing* **38**, 97-139 (2008).

8. Katz K. S., Shutov O., Lapoint R., Kimelman M., Brister J. R.& O'Sullivan C. STAT: a fast, scalable, MinHash-based k-mer tool to assess Sequence Read Archive next-generation sequence submissions. *Genome Biology* **22**, 1-15 (2021).

9. Ondov B. D.*, et al.* Mash: fast genome and metagenome distance estimation using MinHash. *Genome biology* **17**, 1-14 (2016).

10. Apte A.& Daniel S. PCR primer design. *Cold Spring Harbor Protocols* **2009**, pdb. ip65 (2009).

11. Sanger F., Nicklen S.& Coulson A. R. DNA sequencing with chain-terminating inhibitors. *PNAS* **74**, 5463-5467 (1977).

12. Lim S. E.& Copeland W. C. Differential incorporation and removal of antiviral deoxynucleotides by human DNA polymerase γ. *Journal of Biological Chemistry* **276**, 23616-23623 (2001).
